# MARCO, TLR2, and CD14 Are Required for Macrophage Cytokine Responses to Mycobacterial Trehalose Dimycolate and *Mycobacterium tuberculosis*

**DOI:** 10.1371/journal.ppat.1000474

**Published:** 2009-06-12

**Authors:** Dawn M. E. Bowdish, Kaori Sakamoto, Mi-Jeong Kim, Mariliis Kroos, Subhankar Mukhopadhyay, Cynthia A. Leifer, Karl Tryggvason, Siamon Gordon, David G. Russell

**Affiliations:** 1 Sir William Dunn School of Pathology, University of Oxford, Oxford, United Kingdom; 2 Department of Pathology, College of Veterinary Medicine, University of Georgia, Athens, Georgia, United States of America; 3 Department of Microbiology and Immunology, Veterinary Medical Center, Cornell University, Ithaca, New York, United States of America; 4 Division of Matrix Biology, Department of Medical Biochemistry and Biophysics, Karolinska Institutet, Stockholm, Sweden; University of Washington, United States of America

## Abstract

Virtually all of the elements of *Mycobacterium tuberculosis* (Mtb) pathogenesis, including pro-inflammatory cytokine production, granuloma formation, cachexia, and mortality, can be induced by its predominant cell wall glycolipid, trehalose 6,6′-dimycolate (TDM/cord factor). TDM mediates these potent inflammatory responses via interactions with macrophages both *in vitro* and *in vivo* in a myeloid differentiation factor 88 (MyD88)-dependent manner via phosphorylation of the mitogen activated protein kinases (MAPKs), implying involvement of toll-like receptors (TLRs). However, specific TLRs or binding receptors for TDM have yet to be identified. Herein, we demonstrate that the macrophage receptor with collagenous structure (MARCO), a class A scavenger receptor, is utilized preferentially to “tether” TDM to the macrophage and to activate the TLR2 signaling pathway. TDM-induced signaling, as measured by a nuclear factor-kappa B (NF-κB)-luciferase reporter assay, required MARCO in addition to TLR2 and CD14. MARCO was used preferentially over the highly homologous scavenger receptor class A (SRA), which required TLR2 and TLR4, as well as their respective accessory molecules, in order for a slight increase in NF-κB signaling to occur. Consistent with these observations, macrophages from MARCO^−/−^ or MARCO^−/−^SRA^−/−^ mice are defective in activation of extracellular signal-related kinase 1/2 (ERK1/2) and subsequent pro-inflammatory cytokine production in response to TDM. These results show that MARCO-expressing macrophages secrete pro-inflammatory cytokines in response to TDM by cooperation between MARCO and TLR2/CD14, whereas other macrophage subtypes (e.g. bone marrow–derived) may rely somewhat less effectively on SRA, TLR2/CD14, and TLR4/MD2. Macrophages from MARCO^−/−^ mice also produce markedly lower levels of pro-inflammatory cytokines in response to infection with virulent Mtb. These observations identify the scavenger receptors as essential binding receptors for TDM, explain the differential response to TDM of various macrophage populations, which differ in their expression of the scavenger receptors, and identify MARCO as a novel component required for TLR signaling.

## Author Summary

The causative agent of tuberculosis, *Mycobacterium tuberculosis*, has a lipid-rich cell wall that contains a high percentage of mycolic acids. These mycolic acids contribute to both the impermeable nature of the cell wall and to the immunostimulatory properties of the bacterium. Indeed, it has been known for over 50 years that trehalose 6,6′-dimycolate (TDM/cord factor) is the major immunogenic lipid of *M. tuberculosis*, which induces potent pro-inflammatory responses from macrophages, although the receptor has not been identified. We have demonstrated that the toll-like receptor (TLR) pathway is required for pro-inflammatory cytokine production in response to TDM; however, the TLRs alone, or in conjunction with known co-receptors, are not sufficient to induce a response. We demonstrate that the macrophage receptor MARCO, a scavenger receptor, is utilized preferentially to “tether” TDM to the macrophage and activate the TLR2 signaling pathway, and is used preferentially over the related SRA. Macrophages from MARCO^−/−^ mice are defective in activation of TDM-induced signaling and subsequent pro-inflammatory cytokine production in response to both TDM-coated beads and virulent *M. tuberculosis*. By identifying the macrophage receptors involved in initial recognition we can now explain variable responses to TDM between different macrophage populations (which differ in scavenger receptor expression), and have identified a novel co-receptor that may be involved in lipid presentation to TLRs.

## Introduction

*Mycobacterium tuberculosis* (Mtb), a causative agent of human tuberculosis, is responsible for 8 million new infections and 2 million deaths yearly. One third of the world population is currently estimated to be infected with *M. tuberculosis*, although less than 10% of those infected show clinical signs of infection [1]. This is mainly due to the robust granulomatous response that is initiated by the bacterium, which effectively contains the infection and allows the host to exist in equilibrium with a subclinical infection. The granulomatous response has been shown to be triggered by multiple components of the mycobacterial cell wall, such as phosphatidylinositol dimannoside, phosphatidylinositol hexamannoside, and trehalose 6,6′-dimycolate (TDM) [2]–[5].

The chemical structure of TDM (also known as cord factor) was solved in 1956 [6] and was identified as the predominant immunogenic mycobacterial cell wall glycolipid [7]. TDM can elicit pro-inflammatory cytokine production *in vitro*, and granulomatous responses *in vivo*, when administered as a monolayer or part of an oil-water emulsion [7]–[10] . Although it has been known for decades that this pro-inflammatory response is mediated primarily by macrophages, binding or signaling receptors for TDM on macrophages have yet to be identified. Biochemical approaches used in our laboratory to identify TDM receptors, such as affinity isolation using TDM columns, TDM bead phagosome isolation from labeled macrophages, and mass spectrometric analysis of phagocytosed TDM-coated beads after photoactivatable cross-linking, have been unsuccessful. These observations, as well as the finding that the stimulatory activity of TDM requires presentation over a larger surface area, such as emulsions, monolayers, or large diameter particles, suggests that the TDM-receptor interaction is of low avidity and requires the aid of co-receptors or other accessory molecules [9],[10].

One class of receptors implicated in TDM recognition is the TLRs. TLRs have not been shown to bind to TDM directly, but bone marrow-derived macrophages (BMMΦ) from MyD88^−/−^ mice do not produce pro-inflammatory cytokines in response to TDM-coated polystyrene microspheres [9]. Although TLRs have been shown to sense and signal from within the phagosome, they are not phagocytic receptors and usually require the presence of a co-receptor (e.g. CD14) to present their ligands [11].

Class A scavenger receptors (SR), SRA and MARCO, are a class of phagocytic receptors that we have demonstrated mediate recognition and presentation of TDM. SRs bind a range of ligands of endogenous and exogenous origin with relatively low affinity. Ligands for the SRs include proteins [12] and lipids. These lipids can be derived from either the host (e.g. oxidized lipids) [13], or from exogenous sources (e.g. lipopolysaccharide) [14]. Of the two class A SRs, SRA has been clearly demonstrated to be involved in host defense by suppressing excessive pro-inflammatory cytokine production in mouse models of infection and septic shock [14],[15]. MARCO has also been implicated in host defense against bacterial pathogens, but it is not clear whether it is a positive or negative regulator of pro-inflammatory cytokine production [16],[17].

It has been proposed that the class A SRs may be involved in host defense against mycobacterial infection. SRA expression is increased after interferon-gamma (IFN-γ) treatment or exposure to *M. tuberculosis*, and is highly expressed on macrophages associated with *M. bovis* Bacille Calmette-Guérin (BCG)-induced granulomas [14],[18]. There are conflicting reports as to whether expression of SRA increases uptake of *M. tuberculosis* or BCG; however, its presence does not appear to affect the rate of replication of BCG, despite being protective against BCG-primed endotoxic shock [14],[18]. In mouse models, MARCO expression has been shown to be transiently up-regulated on macrophages in response to BCG infection and to be expressed on macrophages within, and adjacent to, BCG-containing granulomas [19]. MARCO-expressing macrophages in the splenic marginal zone appear to phagocytose more BCG than neighboring macrophages that do not express MARCO [19]. The mycobacterial ligands that mediate this recognition have not yet been identified.

Herein, we identify that TDM recognition and signaling is mediated, at least in part, by MARCO, TLR2, and CD14. Although SRA and MARCO have many common ligands, our results show that MARCO binds more TDM-coated beads than either isoform of SRA. MARCO is required for TDM-induced signaling via TLR2 and CD14 in a transfection system, whereas SRAI and SRAII require co-transfection of TLRs 2 and 4, and their accessory molecules, to permit even a minor response to TDM stimulation. Consistent with these data, both resident peritoneal macrophages (RPMφ) and BMMφ from TLR2/4 double-deficient mice (but not the individual mutants) have a markedly reduced response to TDM. This suggests that TDM engages TLR2 and TLR4 in a redundant fashion and that these predominantly MyD88-dependent pathways are required for the stimulatory effects of TDM [9]. When stimulated with TDM-coated microspheres, macrophages from MARCO^−/−^ and MARCO^−/−^ SRA^−/−^ double-deficient (DKO) mice also show reduced activation of ERK1/2 compared to wildtype mice and are defective in subsequent pro-inflammatory cytokine production. These macrophages also produce fewer pro-inflammatory cytokines in response to infection with *M. tuberculosis*, indicating that SR-mediated detection of TDM may be an important component of the response to infection. On the basis of these data we propose a model in which MARCO, and to a lesser extent SRA, cooperate with TLR2 and CD14 for TDM recognition and signaling.

## Results

### Bone marrow-derived macrophages are less responsive to TDM than resident peritoneal macrophages

**Figure 1:**
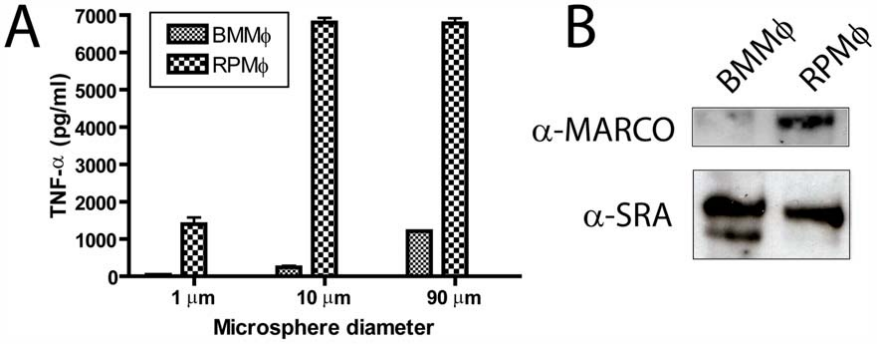
RPMφ are more responsive than BMMφ to TDM-coated microspheres. RPMφ and BMMφ were stimulated with TDM-coated microspheres for 24 h and TNF-α was assayed by ELISA. RPMφ produce substantial levels of TNF-α in response to a range of sizes of TDM-coated microspheres. BMMφ only produce significant amounts of TNF-α in response to large (90 µm diameter) TDM-coated microspheres (Fig. 1A). Bars are representative of three independent experiments; error bars represent the standard deviation of the mean. BMMφ and RPMφ from 129sv/ev mice were collected. SRA is expressed on both BMMφ and RPMφ whereas MARCO is only expressed on RPMφ (Fig. 1B).

BMMφ secrete proinflammatory cytokines in response to TDM coated onto microspheres that are too large to be phagocytosed [9], whereas RPMφ readily produce TNF-α in response to phagocytosable TDM-coated microspheres and secrete much higher amounts than BMMφ in response to larger microspheres (Fig. 1A). RPMφ express high levels of MARCO and SRA, whereas BMMφ express high levels of SRA, but do not express MARCO at levels detectable by immunoblot (Fig. 1B). RPMφ upregulate MARCO at the RNA and protein level in response to TDM-coated 90 µm microspheres, however we have not observed any significant increase in MARCO expression on BMMφ by immunoblot after as many as 72 hours of exposure to LPS or TDM-coated 90 µm microspheres (data not shown). While there are most likely many differences between RPMφ and BMMφ, the absence of MARCO expression on BMMφ may account for the differences in the response to TDM between these two cell types.

### TLR2 and TLR4 are partially redundant for TDM signaling

**Figure 2:**
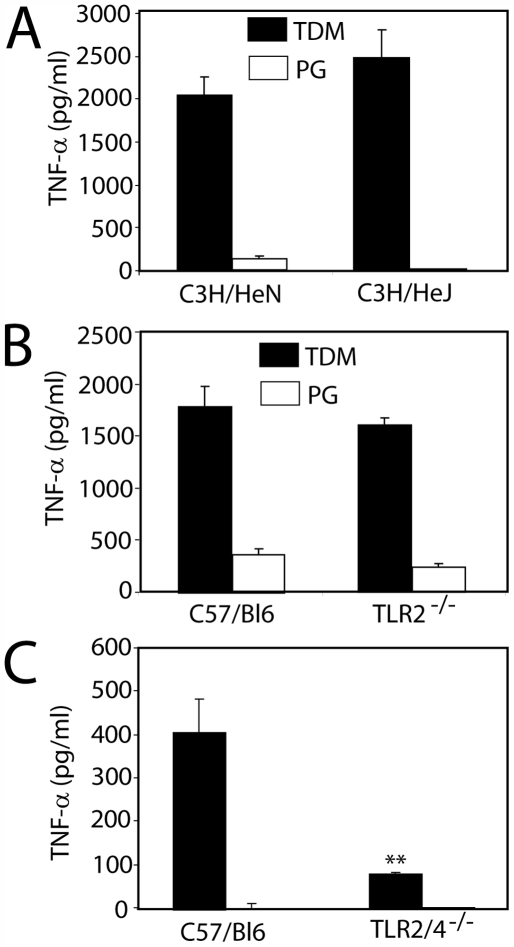
TDM-induced cytokine expression requires either TLR2 or TLR4. BMMφ from mice defective in TLR4 signaling (A) or TLR2 signaling (B) do not have reduced responses to 90 µm TDM-coated beads, however, TLR2/4^−/−^ mice (C) have markedly reduced production of TNF-α in response to TDM, indicating that TLR2 and 4 are redundant for TDM responsiveness. Bars are the mean of triplicate experiments, error bars represent standard deviation of the mean. ** indicates *P*<0.01, as determined by student's *t* test.

We have previously shown that TDM-induced cytokine production is MyD88-dependent but had not identified which TLRs are required (9). Herein we demonstrate that both TLRs 2 and 4 are partially required for TDM-induced cytokine production. BMMφ from C3H/HeJ mice (defective in TLR4 signaling) and TLR2^−/−^ mice showed no reduction in TNF-α production (Fig. 2A&B), while macrophages from TLR2/4^−/−^ mice had greatly reduced TNF-α responses to TDM (Fig. 2C). This suggests that TLR2 and 4 can function at least in a partially redundant manner with respect to TDM responses.

### MARCO, and to a lesser extent SRA, binds to TDM-coated microspheres

**Figure 3:**
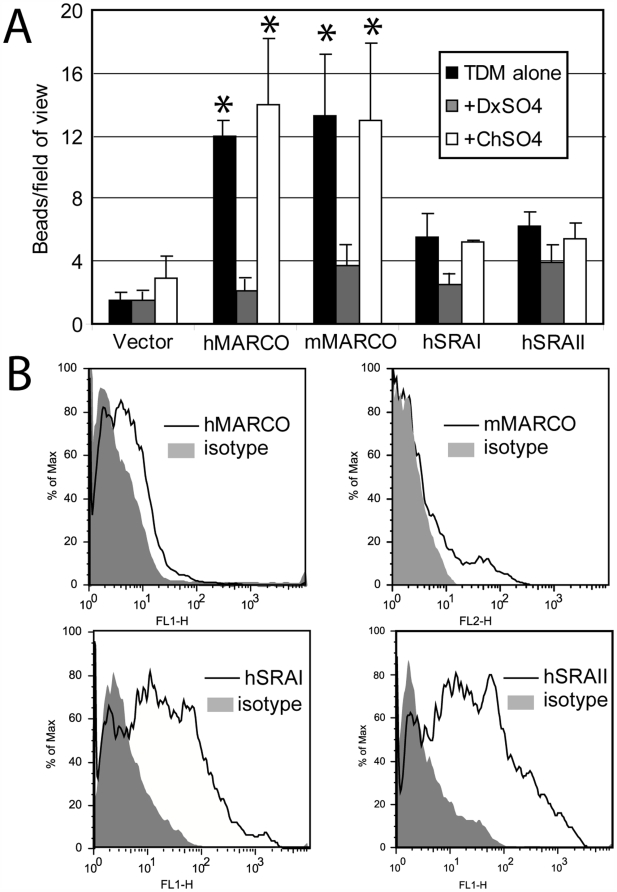
MARCO-transfected cells bind TDM-coated beads. (A) CHO-K1 cells were transfected with plasmids containing human MARCO (hMARCO), mouse MARCO (mMARCO), or two isoforms of human SRA (hSRAI or hSRAII). Binding was assessed by incubating cells on ice, with or without the scavenger receptor inhibitor dextran sulphate (DxSO4) or the non-inhibitor chondroitin sulphate (ChSO4)), and then adding TDM-coated beads. Cells were washed, fixed, and the numbers of beads in at least five fields of view were counted. Bars represent the average of three independent experiments; error bars represent the standard error of the mean. Asterisks indicate *P*<0.05. (B) Immunofluorescence of transfected CHO-K1 cells. Cells were transfected with hMARCO, mMARCO, hSRAI or hSRAII and stained as per the Materials and Methods. SRA expression was consistently higher than MARCO expression. One representative experiment of at least three shown.

In order to determine whether TDM-coated beads were a ligand for MARCO, CHO-K1 cells, which do not express SRs, were transfected with plasmids encoding either MARCO or SRA, and non-opsonic bead binding was assessed. MARCO- and SRA-transfected cells bound significantly more beads than mock-transfected cells, and binding was inhibited by the SR inhibitor dextran sulfate (DxSO4), but not chondroitin sulfate (ChSO4), which does not inhibit the SRs (Fig. 3A). Phosphatidylglyercol (PG) was used as a negative control lipid because we have previously shown that it can be coated onto microspheres in a similar manner and induces a minimal cytokine response from macrophages. PG-coated beads did not bind cells transfected with either MARCO or SRA to a greater extent over empty vector-transfected cells. Furthermore, PG bead binding to cells could not be inhibited by SR inhibitors, indicating that binding was not MARCO- or SRA-specific (data not shown). SRA-transfected cells bound fewer TDM-coated beads despite having higher transfection efficiency (Fig. 3B) indicating that SRA may have a lower binding affinity for TDM-coated beads.

### Expression of MARCO or SRA allows TDM-induced NF-κB signaling in TLR-transfected HEK293 cells

**Figure 4:**
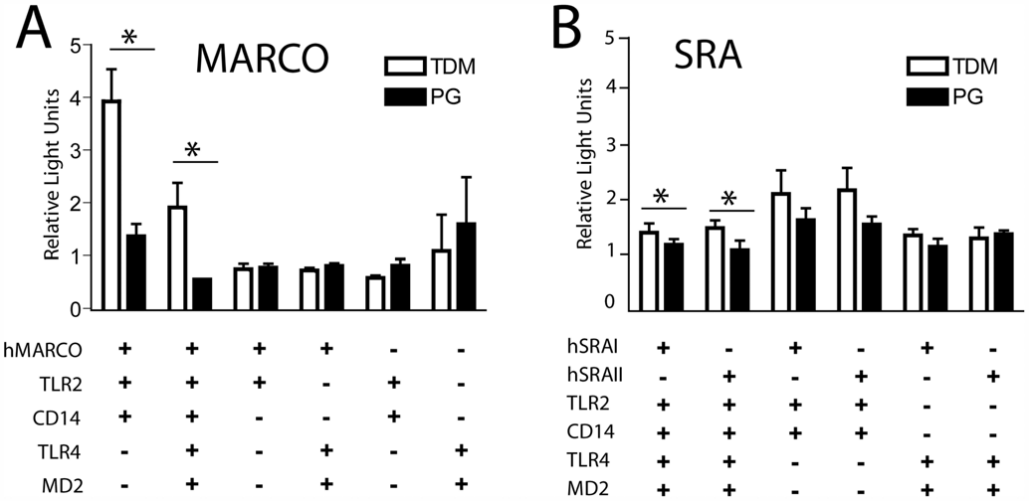
Expression of MARCO restores TLR2-mediated signaling in response to TDM-coated microspheres. HEK293 cells were transfected with plasmids encoding various TLRs (TLR2 or TLR4), accessory molecules (MD2 or CD14), or scavenger receptors (SRAI SRAII, hMARCO), and NF-κB-luciferase and β-galactosidase reporter plasmids. Transfected cells were stimulated with 90 µm diameter TDM- or PG-coated beads, and NF-κB activity (relative light units) was measured. (A) NF-κB activity cannot be induced in transfected cells without, at minimum, hMARCO, TLR2, and CD14. (B) Only a very slight increase in NF-κB signaling is observed when either isoform of SRA is present in addition to TLR2/CD14, TLR4/MD2. PG-coated microspheres do not induce significantly greater NF-κB activation over medium only controls (A and B). Bars are representative of at least 3 independent experiments±standard deviation from the mean. Statistically significant increases (*P*<0.05) are marked with an asterisk.

HEK293 cells do not express most TLRs, except for TLRs 1, 5, and 6 (C. Leifer, personal communication) and are therefore often used for reconstitution assays. By co-transfecting these cells with a luciferase reporter driven by an NF-κB promoter and various combinations of TLRs and SRs, we determined which combinations of receptors allow TDM-induced NF-κB signaling. HEK293 cells transfected with only TLR2 or TLR4, with accessory molecules CD14 or MD2, respectively, did not respond to TDM-coated 90 µm microspheres more than PG-coated microspheres or medium alone (Fig. 4A). The addition of human MARCO (hMARCO) to TLR2/CD14 or TLR2/CD14/TLR4/MD2, however, allowed statistically significant (p<0.05) stimulation of luciferase activity in response to TDM (Fig. 4A). Interestingly, transfection of human SRAI (Fig. 4B) or SRAII (data not shown) required both TLRs 2 and 4, with their respective accessory molecules, in order for TDM-induced stimulation to be observed. These results suggest that MARCO is required for TDM-induced signaling via TLR2/CD14 and that SRA is less efficient in mediating responses to TDM, requiring both TLRs 2 and 4, as well as their accessory molecules.

### TDM- induced binding and signaling is independent of the cytoplasmic region of MARCO

**Figure 5:**
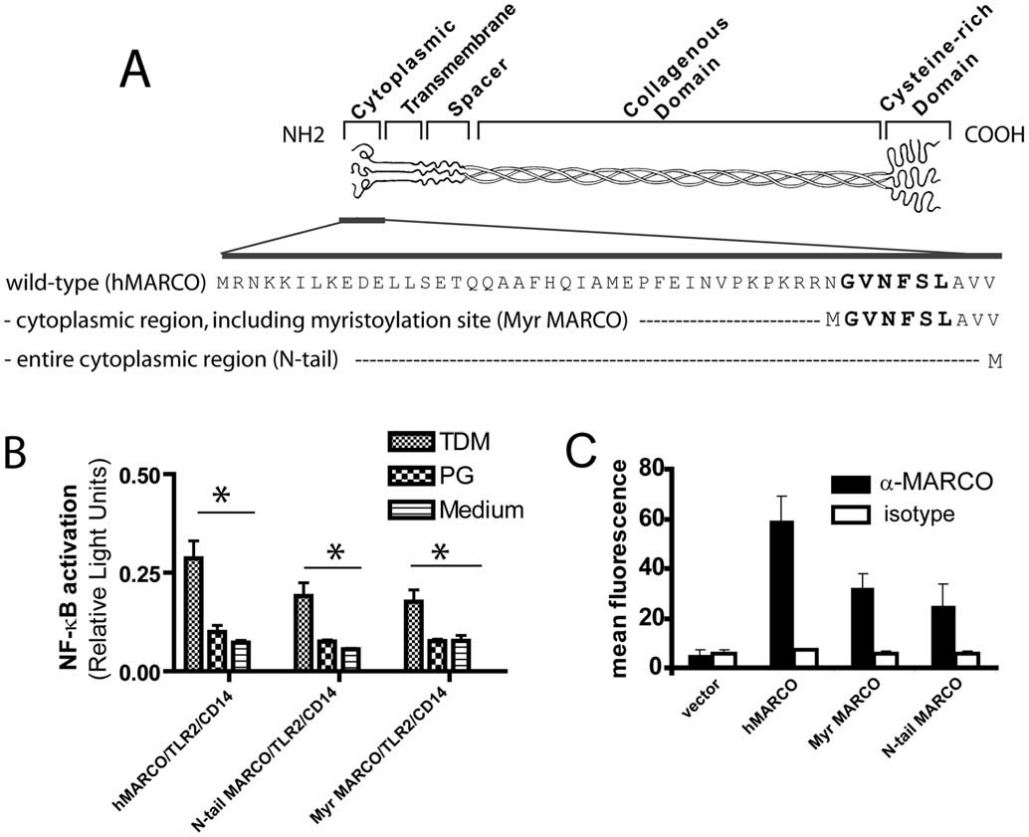
The cytoplasmic region of MARCO is not required for TDM-induced signaling or binding. (A) Constructs of MARCO were cloned. The full length (wildtype) version of MARCO has a 49 amino acid cytoplasmic (NH_2_) tail. Two constructs based on this sequence were made. The first lacks amino acids 1–40, but contains the putative myristoylation site, which is marked in bold (Myr MARCO). The second lacks the entire 49 amino acid cytoplasmic domain (N-tail MARCO). (B) HEK293 cells were transfected with plasmids encoding TLR2, CD14, and the MARCO constructs in addition to the NF-κB-luciferase and β-galactosidase reporter plasmids. Transfected cells were stimulated with 90 µm diameter TDM- or PG-coated beads and NF-κB activity (relative light units) was measured. The presence of full-length or constructs missing all or part of the cytoplasmic tail were sufficient to allow TDM-induced signaling. (C) MARCO constructs are expressed on the surface of HEK293T cells. Although Myr MARCO and N-tail MARCO are expressed at slightly lower levels than hMARCO, they are detectable on the surface of the HEK293T cells as detected by FACS. Bars represent the mean±standard deviation from the mean.

The scavenger receptors have been implicated in modulating TLR signaling by a variety of TLR agonists, but it is not clear if they alter these responses by direct involvement in the signaling pathway. In order to determine whether the cytoplasmic region of MARCO (which would, in theory, transduce signaling events) could contribute to TDM-induced NF-κB activation, two constructs of MARCO were made. The first (Myr MARCO) lacks amino acids 1–40 but contains a putative myristoylation site; the second (N-tail) lacks the entire cytoplasmic domain (1–49) (Fig. 5A). After confirming that the constructs were expressed on the surface of transfected cells (Fig. 5C), their ability to restore TDM-induced NF-κB activation was tested. Cells transfected with either the wild-type MARCO (hMARCO), or the two deletion mutant constructs (Myr MARCO and N-tail MARCO), in addition to TLR2 and CD14, retained their ability to induce NF-κB activation (Fig. 5B). These data suggest that the cytoplasmic region of MARCO is not required for binding or signaling responses to TDM.

### MARCO is recruited to the membrane of TDM-coated bead phagosomes

**Figure 6:**
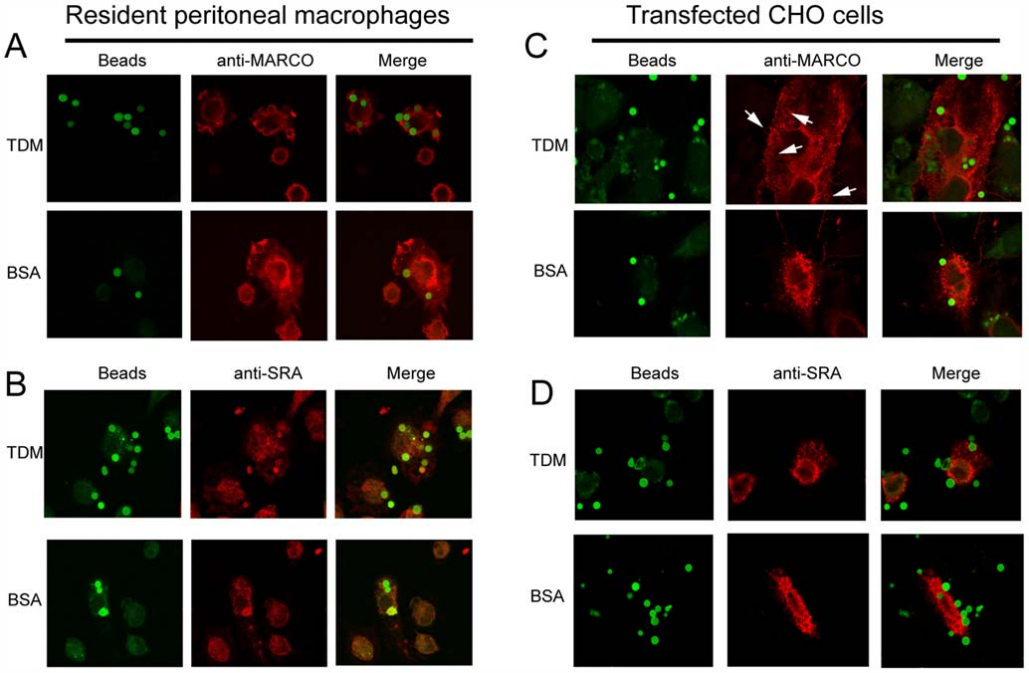
TDM beads induce clustering of MARCO at the phagosomal membrane. RPMφ were stimulated with TDM-coated or negative control (BSA) beads for 5 minutes. (A) Endogenous MARCO is recruited to the phagosomal membrane in response to TDM, but not BSA. SRA staining is apparent at the surface of RPMφ but does not clearly co-localize with the phagosomal membrane surrounding TDM-coated or BSA-coated beads (B). CHO-K1 cells that are transiently transfected with human MARCO stain for concentrated expression around TDM beads (as indicated by arrows) but not BSA-coated beads (C). Transfected human SRA is not clearly recruited to the TDM bead phagosomal membrane to the same extent as MARCO and displays similar diffuse staining in response to TDM-coated beads as BSA-coated beads (D).

Confocal immunofluorescence microscopy was performed to determine whether MARCO or SRA localizes to the membrane of phagosomes containing TDM-coated or control beads. At early time points (5–10 minutes), MARCO recruitment to TDM bead phagosomes was visible in both transfected CHO-K1 cells (Fig. 6C) and RPMφ (Fig. 6A). Recruitment was not clearly evident in MARCO-transfected CHO-K1 cells or in RPMφ that had phagocytosed bovine serum albumin (BSA)- or PG-coated microspheres (Fig. 6A and C, and data not shown). SRA staining of RPMφ did not show strong co-localization with TDM- or BSA-bead phagosomes at this early time point (Fig. 5B), and SRA-transfected CHO-K1 cells showed no appreciable accumulation of SRA at the phagocytic cup in response to either TDM or BSA-coated beads (Fig. 6B and D). At later time points (15–30 minutes), both MARCO and SRA staining occurred at the site of binding to the TDM-, PG-, and BSA-coated microspheres (data not shown), consistent with previous observations that the scavenger receptors are involved in the binding and uptake of polystyrene beads [20]. From these results, we conclude that MARCO is specifically recruited to the phagosomal membrane surrounding TDM-coated beads, whereas SRA recruitment is non-specific.

### Scavenger receptors are required for TDM-induced MAPK activation and TNF-α production

**Figure 7:**
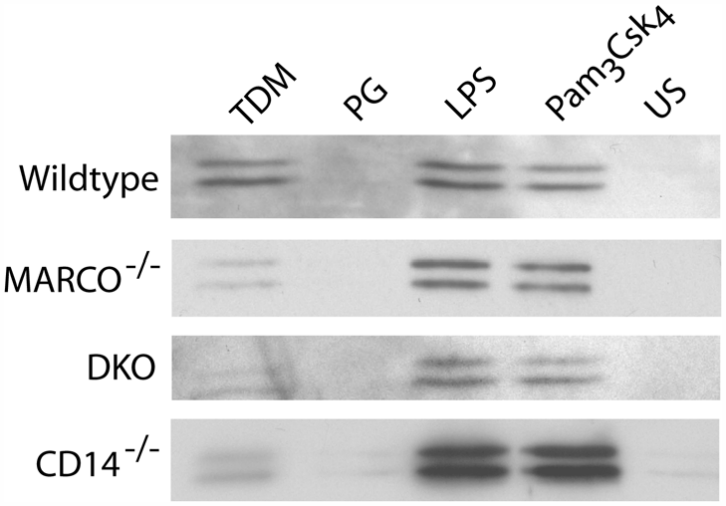
TDM-induced MAPK activation requires scavenger receptors and CD14. TDM induces phosphorylation of ERK1/2 in RPMφ from wildtype mice. Phosphorylation of ERK1/2 is markedly reduced in macrophages from MARCO^−/−^ mice and almost completely abrogated in macrophages from the DKO. A reduction of TDM-induced phosphorylation also occurs in CD14^−/−^ macrophages. Activation of ERK1/2 in response to LPS and Pam_3_Csk_4_ was normal in all knockouts. Blots were stripped and re-probed with antibodies for total ERK1/2 to control for loading differences. One representative experiment of two shown.

RPMφ from wildtype, MARCO^−/−^, SRA^−/−^, MARCO^−/−^SRA^−/−^ (DKO), TLR2^−/−^/TLR4^−/−^ and CD14^−/−^ mice were stimulated with lipopolysaccharide (LPS), Pam_3_Csk_4,_ microspheres coated with either TDM or PG, or medium only, and lysates were immunoblotted for phosphorylated ERK1/2. ERK1/2 was phosphorylated in response to TDM-coated microspheres in wild-type macrophages (Fig. 7). There was no detectable decrease in the TDM response by SRA^−/−^ macrophages (data not shown); however, MARCO^−/−^ macrophages had a reduced response to TDM, and ERK1/2 phosphorylation was further reduced in macrophages from DKO mice (Fig. 7). The levels of ERK1/2 phosphorylation in response to LPS and Pam_3_Csk_4_ were similar between wildtype and SR-deficient macrophages indicating that the SR knockouts do not have a global defect in their ability to phosphorylate ERK1/2 in response to TLR agonists but are specifically defective in the TDM response (Fig. 7). Equivalent levels of ERK1/2 activation by LPS in CD14-deficient RPMφ may be due to soluble CD14 provided by fetal calf serum in the medium and the high dose of LPS used [21].

**Figure 8:**
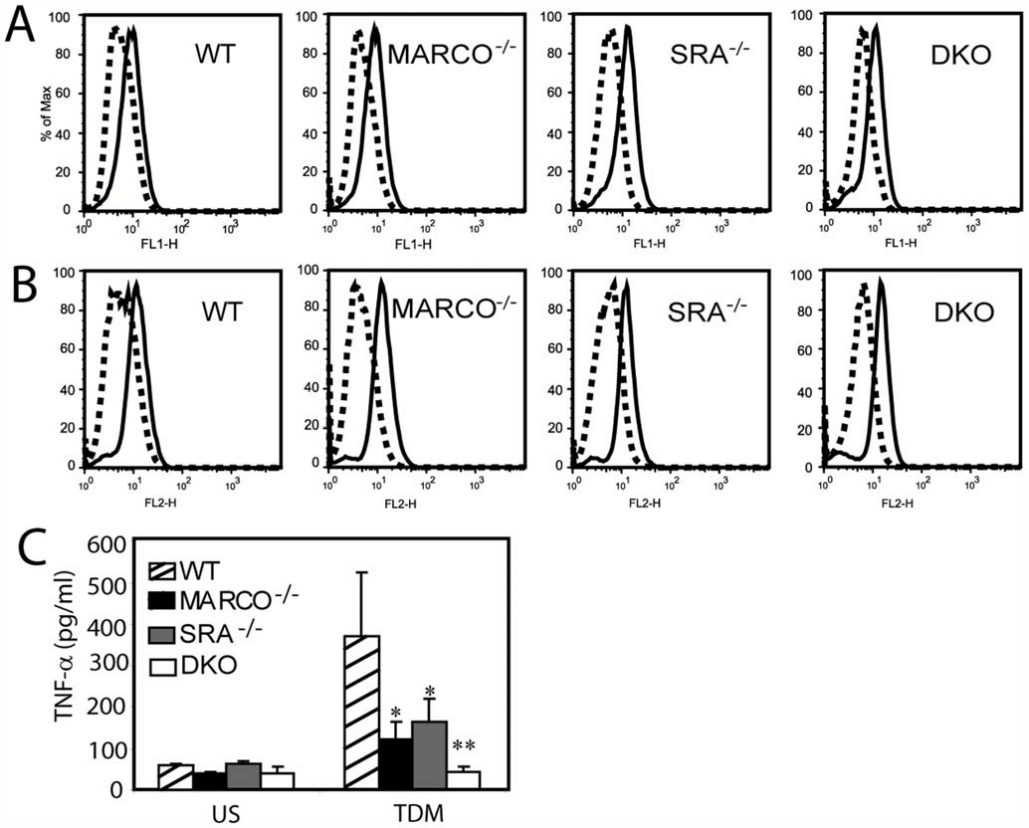
SR knockout mice are defective in responses to TDM. Levels of TLR2 (A) and TLR4 (B) are equivalent between wildtype and SR knockout mice. RPMφ from MARCO^−/−^ and SRA^−/−^ mice demonstrate reduced TNF-α production in response to 3 µM TDM coated beads while macrophages from a DKO are completely abrogated (C). Data from figure C were normalized for cell number using crystal violet staining (see Materials and Methods for details). Bars represent the average of at least three independent experiments; error bars represent the standard error of the mean. Asterisks indicated a significant difference from the wildtype as measured by a student *t* test. * = *P*<0.05, ** = *P*<0.01.

Because the SRs are required for TDM-induced ERK1/2 activation (Fig. 6), we hypothesized that SR-deficient macrophages might also be defective in downstream pro-inflammatory cytokine production. RPMφ from wildtype, MARCO^−/−^, SRA^−/−^ or DKO mice were stimulated with 3 µm diameter TDM-coated beads for 24 hours. Both the MARCO^−/−^ and SRA^−/−^ macrophages had a statistically significant reduction in TNF-α production (*P*<0.05). TNF-α production from DKO macrophages was completely abrogated (*P*<0.025) (Fig. 8C). This is not due to decreased levels of TLR2 (Fig. 8A) or TLR4 (Fig. 8B) expression on the SR-deficient macrophages, as these are both expressed at equivalent levels between wildtype and SR^−/−^ RPMΦ.

**Figure 9:**
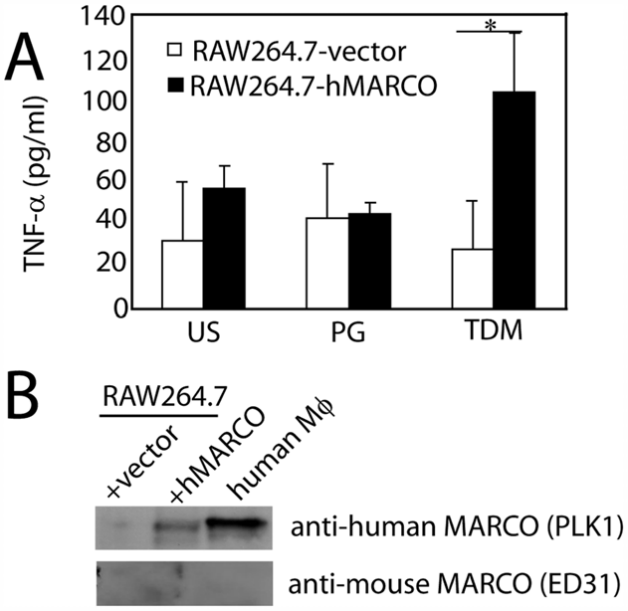
The presence of hMARCO restores TDM responses to a non-responsive cell line. RAW264.7 cells that stably expresses human MARCO (hMARCO- RAW) and a control cell line expressing the empty vector (vector-RAW) were created (B). After 24 hours of stimulation with 3 µm diameter TDM-coated microspheres, the vector-RAW cell line did not produce TNF-α, however there was a significantly increased level of TNF-α production in the hMARCO expressing cell line (A, *P*<0.025). One representative experiment of 3 shown. Bars represent the mean and error bars represent the standard deviation of the mean.

The mouse macrophage cell line, RAW264.7, is similar to BMMφ in expressing high levels of SRA (data not shown) but lacking MARCO at both the protein (Fig. 9B) and RNA levels (data not shown), and in producing lower levels of cytokines in response to TDM as compared to RPMφ (Fig. 9A). We predicted that transfection of RAW264.7 with MARCO could elevate responsiveness to TDM. Stable cell lines expressing either human MARCO (hMARCO-RAW) or an empty vector (vector) were created and stimulated with TDM- or PG-coated microspheres for 24 hours, after which the levels of TNF-α in the medium were assessed by ELISA. Only the hMARCO-expressing cells produced TNF-α in response to the TDM-coated microspheres (*P*<0.05) (Fig. 9A). This result further supports the important role of MARCO in TDM-induced cytokine production.

### MARCO-deficient macrophages do not mount an efficient inflammatory response to *M. tuberculosis*

**Figure 10:**
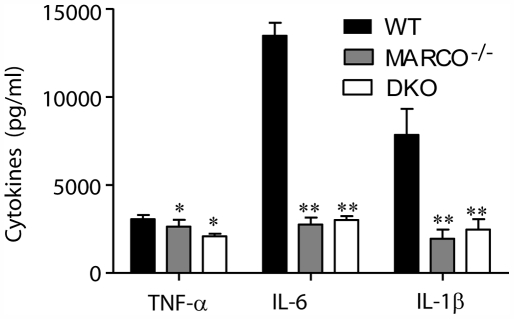
Macrophages from MARCO^−/−^ and DKO mice have significantly reduced cytokine production in response to *M. tuberculosis*. RPMφ were infected with virulent *M. tuberculosis* strain H37Rv for 24 hours. Cytokine production was assessed by ELISA. Macrophages from MARCO^−/−^ and DKO mice were defective in TNF-α, IL-6 and IL-1β. One representative experiment of 3 shown. Bars represent the mean and error bars represent the standard deviation of the mean. Asterisks indicate *P*<0.05(*) or *P*<0.005(**) when compared to wildtype macrophages.

TDM is an essential virulence factor for *M. tuberculosis* pathogenesis and thus we hypothesized that pro-inflammatory cytokine production resulting from *M. tuberculosis* infection would also be impaired in MARCO-deficient macrophages. RPMφ were infected with an MOI of 5 for 24 hours and cytokine production in the supernatants was assessed by ELISA. Consistent with our hypothesis, MARCO^−/−^ and DKO macrophages produced significantly less TNF-α, IL-6, and IL-1β than wildtype macrophages (Fig. 10).

## Discussion

The pathogenesis and establishment of *M. tuberculosis* infection requires phagocytosis of the bacterium by macrophages and initiation of the pro-inflammatory response. These two events are at least partially independent. Phagocytosis is mediated by a number of receptors including the mannose receptor and DC-SIGN which recognize mannose-capped lipoarabinomannan (ManLAM) [22],[23], and complement receptor which mediates the phagocytosis of both opsonized and non-opsonized bacteria [24]. The initiation of a pro-inflammatory response appears to be mediated primarily via TLRs [25] and possibly other signaling receptors such as dectin-1 [26]. Of the mycobacterial cell wall lipids that initiate a TLR-mediated inflammatory response, TDM appears to be one of the more potent [9].

Although we have demonstrated that the macrophage response to TDM is partially TLR2/4-dependent (Fig. 2), our initial attempts to reconstitute NF-κB signaling in a TLR2/4 stably-transfected cell line were not successful. It seemed likely that this was due to the requirement of an additional co-receptor, because many TLR ligands, and especially lipid-based ligands, require presentation via a co-receptor. The co-receptor CD14 has been implicated in facilitating TLR1/2-mediated responses to bacterial lipopeptides by enhancing the physical proximity of the ligand to the TLR1/2 heterodimers, without binding directly to the receptor complex [25],[27],[28]. However, CD14 expression in conjunction with TLR2 or TLR4 did not restore responsiveness to TDM (Fig. 4) suggesting that CD14 was not the only co-receptor for TDM. Because MARCO and SRA bind to TDM-coated beads (Fig. 3A), we hypothesized that they might be the additional co-receptors required for TLR2 signaling. Both MARCO and CD14 in conjunction with a TLR2 homo- or heterodimer appear to be required to initiate TDM signaling, and MARCO appears to be preferred over the closely related SRA (Fig. 4A and B, Fig. 7). We therefore propose that the scavenger receptors function as co-receptors that, in conjunction with CD14, present TDM to TLR2. Further work is warranted to determine whether TDM signaling requires an additional receptor such as TLR1 or TLR6. The structure of TDM, however, does not include di-acylated lipids that signal via TLR2/6 heterodimers or tri-acylated lipids that signal via TLR1/2 heterodimers [29].

**Figure 11:**
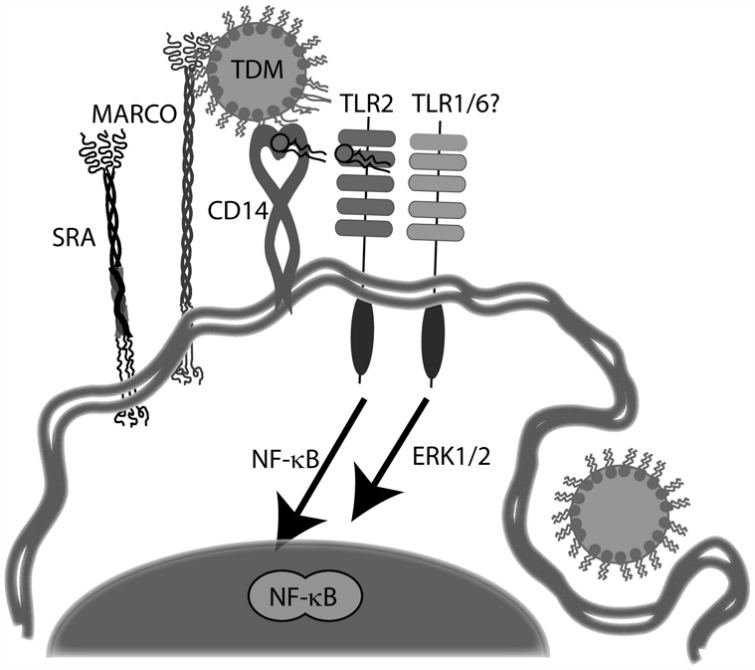
MARCO is a component of the TDM signaling complex. MARCO functions as a tethering receptor that functions in conjunction with CD14 and TLR2. Presumably CD14 is involved in extracting or presenting the lipid to the TLRs. MARCO is used preferentially over SRA, possibly because it has a higher binding affinity for TDM. Phagocytosis occurs concurrently but is not necessarily mediated by the class A scavenger receptors.

SRA has been demonstrated to modulate TLR signaling [30],[31] and macrophages from both SRA- and MARCO-deficient mice have skewed cytokine responses in response to TLR agonists [16]. It is not entirely clear, however, if the SRs are able to signal directly or if they have an indirect function in the signaling pathway, for example, by phagocytosing and clearing TLR agonists. In order to test whether MARCO might be signaling directly, we created mutants that lacked the cytoplasmic domain, and thus any putative signaling motifs, of MARCO. The cytoplasmic region of MARCO has not been experimentally demonstrated to have any residues that are essential for cell signaling and indeed, apart from the putative myristoylation site at residues 41–46, does not contain any potential signaling motifs identifiable by scanning various protein motif databases (D.M.E.Bowdish, unpublished results). After confirming that these constructs were expressed on the surface of transfected CHO-K1 and HEK293 cells, we determined that they could indeed bind to TDM-coated beads and were in fact able to reconstitute TDM-induced NF-κB signaling (Fig. 4). Our receptor-ligand interaction assay results, however, show that MARCO alone is not sufficient for TDM binding, and that other receptors present on the surface of RPMφ must cooperate with MARCO for effective binding to occur (Fig. S1, Text S1). Signaling induced by TDM may be independent of the phagocytic function of the scavenger receptors, as macrophages from the SRA^−/−^, MARCO^−/−^ and DKO mice did not have any detectable defect in phagocytosis of TDM-coated beads (data not shown). These data are consistent with a role for MARCO as a “tethering” receptor for TDM, perhaps extracting individual lipids and presenting them to the CD14/TLR2 complex (Fig. 11), but MARCO itself appears to lack a direct signaling function.

Our observation that MARCO is the preferred receptor for TDM may explain why some macrophage populations respond robustly to TDM, while others do not. For example, RPMφ, which express high levels of MARCO, respond strongly to TDM, whereas BMMφ and RAW264.7 cells, which express SRA but not MARCO, produce a minimal amount of pro-inflammatory cytokines in response to TDM. We therefore propose that MARCO is the preferred receptor for TDM, although the less avid interaction between SRA and TDM can also facilitate signaling through TLRs. This hypothesis is consistent with the work of Ozeki *et al.* in which it was shown that SRA can bind to TDM *in vitro* and plays a role in suppressing TNF-α production by alveolar macrophages or Kupffer cells in response to TDM-coated wells [32].

Because TDM is highly immunogenic, it is being studied as an adjuvant that boosts both humoral and cellular immune responses [33],[34], is a novel candidate for vaccine development [35], and is used to mimic the pathogenesis of *M. tuberculosis* infection [36]. Indeed, our observation that MARCO-deficient macrophages are defective in pro-inflammatory cytokine production in response to either TDM or virulent *M. tuberculosis* is consistent with our hypothesis that TDM is the major immunogenic lipid associated with pro-inflammatory responses. It is likely that TDM is not the only ligand for the scavenger receptors on *M. tuberculosis* and further study is warranted to elucidate what these interactions might be; however, our data suggests that this interaction is a major component of the macrophage response to infection. We propose that TDM is a novel SR ligand that binds to MARCO with a higher affinity than SRA and that the TDM-induced pro-inflammatory response is mediated in large part via SR/TLR2/CD14 receptors. . These observations may explain why some macrophage populations respond more strongly than others to TDM and thus will provide novel insight into the role of the scavenger receptors in the pathogenesis of tuberculosis.

## Materials and Methods

### Animals

C57BL/6, SRA^−/−^, MARCO^−/−^, MARCO^−/−^ SRA^−/−^ (DKO), CD14^−/−^, C3H/HeN, C3H/HeJ and 129sv/ev mice were bred and housed at the University of Oxford, Cornell University, or University of Georgia. All SR knockout mice were created on the C57BL/6 background strain [15],[17],[37]. The SRA^−/−^ and DKO mice are deficient in SRAI and SRAII. Unless otherwise stated, C57BL/6 mice were used as wild-type and were purchased from either Taconic or Harlan. TLR2/4 double-deficient mice were created by Shizuo Akira (Osaka University), generously supplied by Lynn Hajjar (University of Washington), and were bred at the Cornell University Transgenic Mouse Core Facility. All mice were housed in specific pathogen-free conditions and experiments were designed to use age- and sex-matched mice, between 5 weeks and 3 months of age. All animal experiments were approved by the ethics board of the university at which the experiments were performed (i.e. University of Oxford or Cornell University).

### Cell culture

CHO-K1 cell line (ATCC#CCL-61) was maintained in Ham's F12K medium (Gibco) supplemented with 10% heat-inactivated fetal calf serum (HI-FCS, Hyclone), 2 mM L-glutamine, 1.5 g/L sodium bicarbonate, 100 U/ml penicillin, and 100 µg/ml streptomycin (Gibco). CHO-K1 cells were transfected using Lipofectamine as per the manufacturer's instructions (Invitrogen). The HEK293 cell line (ATCC# CRL-1573) was provided by Cynthia Leifer, cultured in Dulbecco's Modified Eagle Medium (DMEM; Gibco) supplemented with 10% HI-FCS, 2 mM L-glutamine, 1 mM sodium pyruvate, 10 mM HEPES (Gibco), 100 U/ml penicillin, and 100 µg/ml streptomycin. Transfected RAW 264.7 cells were maintained in RPMI, 10% HI-FCS, 2 mM L-glutamine, and 100 µg/ml ascorbic acid (Sigma). BMMφ were cultured as described previously [9] and maintained in DMEM supplemented with 20% L-929 cell-conditioned media, 10% HI-FCS, 2 mM L-glutamine, 1 mM sodium pyruvate, 100 U/ml penicillin, and 100 µg/ml streptomycin. RPMφ were obtained by lavaging the peritoneal cavity with 10 ml cold phosphate-buffered saline (PBS), re-suspending the peritoneal cells in complete growth medium (DMEM supplemented with 10% HI-FCS, 2 mM L-glutamine, 1 mM sodium pyruvate, 100 U/ml penicillin, and 100 µg/ml streptomycin) and allowing the macrophages to adhere to Petri dishes overnight. Non-adherent cells were then rinsed off before RPMφ were used for experiments. All media components were routinely tested for endotoxin by Limulus Amoebocyte Assay (Cambrex). All cells were maintained at 37°C and 5% CO_2_. .

### Antibodies and other reagents

The monoclonal anti-MARCO clones ED31 (anti-mouse MARCO) and PLK-1 (anti-human MARCO), and anti-SRA clone 2F8 were grown and maintained at the Gordon laboratory. Polyclonal anti-hMARCO antibodies were a generous gift from Dr. Timo Pikkarainen. Anti- mouse TLR2 and TLR4 antibodies were purchased from eBioscience. Rabbit antibodies for phosphorylated and total ERK1/2 (Cell Signaling Technologies) were used according to manufacturer's protocols. Peroxidase-conjugated goat anti-mouse or anti-rabbit antibodies (Jackson Labs) were used at 1∶200 for immunoblotting. AlexaFluor 488 or 594 goat anti-mouse IgG antibodies (Molecular Probes) were used as secondary antibodies for immunofluorescence.

TDM from *M. tuberculosis* H37Rv strain and bovine-derived PG (Sigma-Aldrich) were resuspended in chloroform/methanol (2∶1 v/v) at 10 mg/ml and stored at −20°C under nitrogen. Sterile 3 µm and 90 µm diameter polystyrene microspheres or 2.5 µm fluorescent polystyrene microspheres (Polysciences) were coated with TDM or PG as described previously [9]. Coated microspheres were washed and re-suspended in endotoxin-free PBS (Invitrogen) at 2% solids. It should be noted that the manufacturing protocol for these microspheres was changed by Polysciences in 2007, resulting in less efficient coating of the polystyrene particles by lipids and therefore reduced immunostimulatory ability. Original results could be reproduced using 80 µm diameter polystyrene microspheres from Duke Scientific.

### Plasmids and constructs

Human TLR4, MD2, TLR2, and CD14 plasmids were generously provided by Dr. Cynthia Leifer (Cornell University). All plasmids were amplified and purified using Endo-free Maxi Prep columns (Qiagen). The plasmids containing human MARCO (hMARCO), mouse MARCO (mMARCO), human SRAI and SRAII have been previously described [38],[39]. Constructs of human MARCO that were missing the cytoplasmic tail (N-tail hMARCO or Myr hMARCO) were created by designing primers that amplified the transmembrane region of hMARCO and contained restriction enzyme sites. After amplification and restriction digest, the amplified fragment was sub-cloned into pcDNA3.1 (Invitrogen).

Stable cell lines expressing either hMARCO or the empty vector (pcDNA3) were created in RAW264.7 cells by transfecting the cells per the manufacturer's directions (GeneJuice, EMDbiosciences) and selecting under G418. Surviving cells were tested for MARCO expression by flow cytometry and immunoblot, and were cultured in 1 mg/ml G418 until use.

### Microsphere binding assay

CHO-K1 cells were transfected with plasmids encoding either MARCO or SRA as described above. Medium was removed and replaced with Opti-MEM (Invitrogen). Non-opsonic bead binding was assessed by incubating the cells on ice for 30 min with or without inhibitors, then adding TDM or PG-coated, fluorescent, 3 µm diameter microspheres. After 30 min on ice, the cells were washed, fixed, and bead binding was assessed by microscopy. Dextran sulfate (DxSO_4_) or chondroitin sulfate (ChSO_4_) were used at concentrations of 100 µg/ml.

### Luciferase assay

HEK293 cells were seeded at 5×10^5^ cells per well in 2 ml of medium per well in a 6 well plate overnight. HEK293 cells were transfected according to manufacturer's protocol using TransIT transfection reagent (Mirus) with 144 ng each of NF-κB-luciferase and β-galactosidase reporter plasmids, 30 ng each of TLR2, CD14, or TLR4, 90 ng MD2, and 300 ng MARCO or SRAI/II per well depending on the experiment. Total DNA was brought to 2 µg using empty vector (pcDNA3.1). Transfected cells were incubated for 24 hours before trypsinization and reseeding in 96 well plates (one row of a 96 well plate from each well of the 6 well plate) in 100 µl media/well. After another 24 hours, cells were stimulated with either 1.25×10^3^ TDM- or PG-coated 90 µm polystyrene microspheres per well, or positive control ligands 1 µg/ml Pam_3_Csk_4_ (Calbiochem) or 100 ng/ml lipopolysaccharide (Sigma) as positive controls for TLR2 and TLR4, respectively. Only data from experiments, in which positive responses to LPS and Pam_3_Csk_4_ were consistent between relevant transfectants, were used in order to assure functional TLR4 and TLR2 complexes were expressed at equivalent levels. In addition, only experiments in which equivalent levels of MARCO and SRA were expressed, as determined by FACS, were used. After 18 hours, transfected cells were lysed using Reporter Lysis Buffer (Promega) and lysates were analyzed for luciferase (Promega) and β-galactosidase (Tropix) activity using a Veritas luminometer (Turner Biosystems). NF-κB activity (relative light units) was measured by dividing luciferase activity by β-galactosidase activity, and then fold activity was calculated by dividing TDM and PG results by medium only results. Statistical significance was determined using Student's *t* test.

### Immunofluorescence and FACS

RPMφ, or CHO-K1 cells transfected with hMARCO for 24 hours as described above, were seeded onto cover slips at 1×10^5^ cells per well in 500 µl of media. Washed, 3 µm diameter, carboxylated silica beads (Kisker Biotech) were re-suspended in 25 mg/ml of the heterobifunctional crosslinker cyanimide and incubated with agitation for 15 minutes. Excess cyanimide was removed by washing twice in 0.1 M sodium borate pH 8.0 (Sigma-Aldrich). The beads were then cross linked to defatted BSA in a 10 mg/ml solution for 2 hours with agitation, followed by labeling with 2 µg/ml carboxyfluoresceinthiosemicarbazide (Molecular Probes) for 30 minutes with agitation. After washing with PBS, the beads were then passively coated with TDM as described above. Beads were added to cells at a ratio of 5∶1, maintained at room temperature for five minutes, then incubated at 37°C for five minutes, before rinsing with PBS and fixing in 4% paraformaldehyde in PBS. Cells were blocked overnight at 4°C in staining buffer (SB; 1% BSA, 1% heat-inactivated goat serum, 0.25% saponin in PBS), then incubated with 10 µg/ml primary antibody in SB for 1 hour at room temperature, washed three times with PBS, and then incubated with secondary goat anti-mouse antibody also for 1 hour at room temperature. Cover slips were rinsed three times with PBS, then quickly in double-distilled water before mounting using Prolong Gold Antifade medium (Molecular Probes). Confocal images were taken using an Olympus Fluoview 500 confocal laser scanning imaging system equipped with argon, krypton, and He-Ne lasers on an Olympus IX70 inverted microscope with a PLAPO 60× objective (Olympus America, Inc.). Confocal images were processed using Adobe Photoshop 6.0 (Adobe Systems, Inc.,).

For FACS analysis, cells were transfected with plasmids as described above and stained with either the appropriate MARCO- or SRA-specific antibodies or corresponding isotype controls as per standard protocols.

### Immunoblotting

To assess SR expression, BMMφ, RPMφ, and RAW264.7 cells were seeded in 6 well plates overnight in the appropriate media (discussed above). The cells were lysed in RIPA buffer [50 mM Tris-HCl (pH 7.4), NP-40 1%, sodium deoxycholate 0.25%, NaCl 150 mM, EDTA 1 mM, PMSF 1 mM, and protease inhibitors (Roche)], then assessed for protein concentration by Bradford Assay (Bio-Rad). Equivalent amounts of protein per sample were boiled in 2× non-reducing sample buffer for five minutes, centrifuged, and separated by sodium dodecyl sulfate-polyacrylamide gel electrophoresis (SDS-PAGE). Samples were transferred to nitrocellulose membranes, and then blocked in 5% nonfat dry milk in 0.1% Tween-20 in PBS (PBST) overnight with gentle rocking. Both primary and secondary antibodies were diluted in blocking buffer, and applied to membranes for one hour at room temperature with gentle rocking, and with three 5 minute washes with PBST between incubations.

For MAPK immunoblots, RPMφ were seeded at approximately 5×10^5^ cells per well in a 24 well plate in R10 medium and allowed to adhere overnight. After 16 hours, the cells were washed once with PBS and the medium was replaced with 450 µl of warm R10. RPMφ were stimulated with either 100 ng/ml of LPS, 1 µg/ml Pam_3_Csk_4_, or 1.9×10^3^ TDM- or PG-coated 90 µm diameter beads, or PBS as a vehicle control. After stimulation, the medium was removed and the cells were lysed using 200 µl of hot 2× reducing sample buffer. Lysates were placed on ice, syringed to shred DNA, then boiled for five minutes at 100°C, centrifuged and stored at −20°C until use. Equal volumes of sample were loaded per lane for SDS-PAGE. Proteins were transferred to nitrocellulose membranes and blocked with 5% nonfat dry milk in TBST [10 mM Tris–HCl (pH 8), 150 mM NaCl, 0.1% Tween-20]. The filters were then incubated overnight at 4°C with anti-ERK1/2-P antibodies (Cell Signaling Technology). Anti-total ERK1/2 antibody was used to show equivalent lane loading. Immunoreactive bands were detected using horseradish peroxidase-conjugated goat anti-rabbit IgG antibodies (Jackson Immunoresearch) and chemiluminescence (Pierce).

### Cytokine assay

BMMφ or RPMφ were counted and seeded at approximately 1×10^5^ cells/well in 100 µl media/well in 96 well plates, or 4×10^5^ cells/well in 500 µl media/well in 24 well plates. RAW264.7 constructs were counted and seeded at approximately 1×10^4^ cells/well in 96 well plates. RPMφ were washed once with PBS to remove non-adherent cells and the media replaced with Opti-MEM. Macrophages were stimulated with either TDM or PG-coated microspheres, with dose normalization for bead surface area (total bead surface area of 5×10^7^ µm^2^/well), 100 ng/ml of LPS (*E. coli*, Sigma-Aldrich), or 100 ng/ml Pam_3_Csk_4_ (Calbiochem); PBS was added as a vehicle control. After 24 h, the media were collected and the concentration of TNF-α was determined by ELISA as per the manufacturer's directions (BD Bioscience or eBioscience). In experiments comparing RPMφ from different mouse genotypes, cells were stained with crystal violet to normalize for cell numbers. The crystal violet was solubilized with 1% SDS and the resulting supernatant was read on a plate reader (OD 550 nm). TNF-α release is expressed as pg/ml/OD550 to normalize for cell numbers.

### In vitro infection of RPMφ

RPMφ were collected from MARCO^−/−^, SRA^−/−^, and DKO mice as described above and seeded in equivalent numbers in 24 well plates, depending on the genotype with the lowest yield (approximately 1×10^5^ cells per well). Mid-log phase cultures of Mtb H37Rv strain were washed then added to the cells at an MOI of 5 in 250 µl of medium. After 24 hours the medium was filtered and analyzed for cytokines by ELISA.

### Statistical analysis

Differences between the means of experimental groups were analyzed using Student's *t* test. Differences were considered significant when *P*≤0.05.

## Supporting Information

Figure S1 TDM requires other receptors in order to pull down MARCO. Radiolabelled hMARCO was added to TDM covalently-linked to Carbolink (lane 1), fucoidan-Carbolink (lane 2), or Carbolink only (lane 3). The positive control shows the radioactive signal of 1 µl of the original radiolabelled hMARCO mixture. Only fucoidan-Carbolink pulls down hMARCO under these conditions. When RPMφ lysates are also added to TDM-Carbolink (lane 4) or Carbolink only (lane 5) pulldowns, both pull down small amounts of hMARCO, with slightly more bound to TDM. Lanes 4 and 5 were adjusted for greater contrast using ImageQuant software (Molecular Dynamics).
(1.44 MB TIF)

Text S1 Supplementary materials and methods.
(0.03 MB DOC)
