# Supplementary figures and images for: MARCO, TLR2, and CD14 Are Required for Macrophage Cytokine Responses to Mycobacterial Trehalose Dimycolate and Mycobacterium tuberculosis
Source: PLoS Pathog. 2009 Jun 12;5(6):e1000474. doi: 10.1371/journal.ppat.1000474 (PMC2688075; doi:10.1371/journal.ppat.1000474)

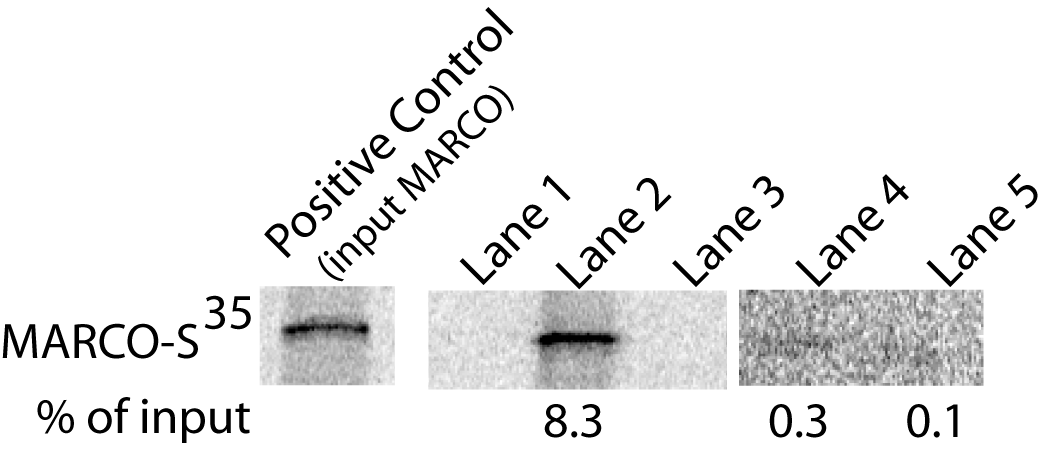

Supplement: Figure S1 — TDM requires other receptors in order to pull down MARCO. Radiolabelled hMARCO was added to TDM covalently-linked to Carbolink (lane 1), fucoidan-Carbolink (lane 2), or Carbolink only (lane 3). The positive control shows the radioactive signal of 1 µl of the original radiolabelled hMARCO mixture. Only fucoidan-Carbolink pulls down hMARCO under these conditions. When RPMφ lysates are also added to TDM-Carbolink (lane 4) or Carbolink only (lane 5) pulldowns, both pull down small amounts of hMARCO, with slightly more bound to TDM. Lanes 4 and 5 were adjusted for greater contrast using ImageQuant software (Molecular Dynamics). (1.44 MB TIF) [file ppat.1000474.s001.tif]
