# Supplementary material for: MARCO, TLR2, and CD14 Are Required for Macrophage Cytokine Responses to Mycobacterial Trehalose Dimycolate and Mycobacterium tuberculosis
Source: PLoS Pathog. 2009 Jun 12;5(6):e1000474. doi: 10.1371/journal.ppat.1000474 (PMC2688075; doi:10.1371/journal.ppat.1000474)
Supplement: Text S1 — Supplementary materials and methods. (0.03 MB DOC) [file ppat.1000474.s002.doc]

**Online Supplemental Material**

**In vitro receptor-ligand interaction assays**

2.5 mg lyophilized *M. tuberculosis* H37Rv TDM (Sigma-Aldrich) or fucoidan (Fluka BioChemika) were dissolved in 1 ml of coupling buffer (CB; 0.1 M NaH_2_PO_4_) with 0.5 % TritonX-100 (Sigma-Aldrich). The solution was sonicated until uniformly cloudy. 4.5 mg of sodium meta-periodate (Sigma-Aldrich) was added to the solution, followed by gentle mixing for 30 minutes at room temperature. One ml of Carbolink (Pierce) per sample was washed five times with CB, then 500 μl of the TDM or fucoidan mixture was added, and the tubes were covered and mixed overnight at room temperature. The covalently-linked Carbolink samples and a Carbolink only sample were then washed five times with PBS, followed by washing five times in modified Latz lysis buffer [LLB; 10 mM Tris-HCl (pH 7.4), 137mM NaCl, and 0.5% Triton X-100]. Radiolabelled MARCO was produced using the TNT T7 Quick Coupled Transcription/Translation System (Promega) and Redivue L-[^35^S]methionine (Amersham Biosciences) according to the manufacturer‘s protocols. Seven μl of the radiolabelled human MARCO TNT reaction were added to one ml of LLB with 1 mM MgCl_2_ and 2 mM CaCl_2_, containing 25 μl covalently-linked Carbolink or Carbolink only, with or without cell lysates. For cell lysates, confluent 90 mm^2^ petri dishes of C57BL/6 RPMφ, differentiated THP-1 cells, or six well plates of HEK293 cells transfected with human CD14 (as described above), were rinsed with PBS, then incubated at 4^o^C in PBS for 10 minutes, scraped and spun at 200 g for five minutes at 4^o^C. The RPMφ, THP-1, or HEK293 pellet was then resuspended in two, two, or one ml, respectively, of LLB containing Protease Inhibitor Cocktail III (Calbiochem), 1 mM PMSF, 1 mM MgCl_2_, and 2 mM CaCl_2_, and rocked with agitation for one hour at 4^o^C. The various assay mixtures were mixed overnight at 4^o^C. The samples were then washed twice with LLB containing divalent cations, with two minute incubations on ice between washes. The pellets were resuspended in 20 μl of 2X non-reducing sample buffer, boiled for two minutes, centrifuged, and stored at -20^o^C until use. Samples were run on 10% SDS-PAGE gels, dried onto filter paper (Whatman), and exposed to a phosphor screen (Kodak) overnight. Radioactive bands were detected using a Storm 860 scanner (GE Healthcare) and analyzed using ImageQuant (Molecular Dynamics) software. Per cent input was calculated as: (sum of band signal intensity – sum of background intensity)/ 7 μl radiolabelled hMARCO as a per cent of (sum of input band signal intensity – sum of background intensity).
